# The effect of frailty on the adjustability of grasping force in community-dwelling older adults: a cross-sectional study

**DOI:** 10.1186/s12877-025-06428-0

**Published:** 2025-11-13

**Authors:** Tatsuya Kaneno, Akihiro Sato, Kazunori Akizuki, Jun Yabuki, Satoshi Shibata, Yoshifumi Morita

**Affiliations:** 1 Department of Occupational Therapy, Graduate School of Human Health Sciences, Tokyo Metropolitan University, 7-2-10 Higashi-Ogu, Arakawa-ku, Tokyo, 116-8551 Japan; 2 Department of Occupational Therapy, Mejiro University, Saitama, Japan; 3 Department of Physical Therapy, Mejiro University, Saitama, Japan; 4 Department of Physical Therapy, Ibaraki Prefectural University of Health Sciences, Ibaraki, Japan; 5 Graduate School of Engineering, Nagoya Institute of Technology, Nagoya, Japan

**Keywords:** Aged, Independent living, Frailty, Hand strength

## Abstract

**Background:**

This study aimed to clarify the influence of frailty on the adjustability of grasping force (AGF) in community-dwelling older adults.

**Methods:**

This cross-sectional study included 23 community-dwelling older adults. Participants were assessed for basic information, the Japanese version of the Cardiovascular Health Study (J-CHS) criteria, the Frenchay Activities Index, and AGF. The participants were classified into the robust and non-robust groups based on the J-CHS criteria. AGF was quantified using the AGF score, defined as the absolute error between the target and actual grasping force; lower AGF scores indicated better AGF.

**Results:**

The non-robust group showed significantly lower AGF scores than the robust group in the total (*p* = 0.02), isometric (*p* = 0.04), and concentric (*p* = 0.01) sections of the non-dominant hand, indicating better AGF. In the robust group, AGF scores in the concentric section differed significantly between the dominant and non-dominant hands (*p* = 0.02), with the dominant hand showing a lower AGF score, indicating better AGF. In the non-robust group, AGF scores in the isometric section differed significantly between the dominant and non-dominant hands (*p* = 0.04), with the non-dominant hand showing a lower AGF score, indicating better AGF.

**Conclusions:**

These findings suggest that non-robust older adults may compensate for functional limitations by enhancing AGF in the non-dominant hand.

## Introduction

In 2023, 29.1% of Japan’s population was aged ≥ 65 years, and this proportion continues to rise [1]. As the population ages, the demand for long-term care will increase, along with the number of older adults unable to live independently in the community [2]. Frailty, a key reason for long-term care, accounts for 13.8% of cases and affects individuals even in the absence of specific diseases [2]. Frailty is characterised by reduced physiological reserves and increased vulnerability to various stressors associated with ageing [3, 4]. Hand function is crucial for activities of daily living (ADL) and plays a role in reaching, pinching, catching, picking, and grasping. Consequently, research on hand function in older adults has gained attention, with studies highlighting the importance of assessing characteristics of hand function [5, 6].

Various methods for assessing hand function are used in clinical settings [7–11]. However, most existing methods evaluate hand function based on the task completion time or success rate, which captures only one aspect of hand function. Practical grasping movements require adjustability in grasping force according to the shape, weight, and material of an object (i.e., the adjustability of grasping force [AGF]), in addition to maximal performance such as grip strength. We have studied the AGF [12–14]; in a study comparing the AGF between older and younger adults, older adults exhibited poorer AGF, indicating the effect of ageing [14]. However, no studies have examined AGF differences between robust and non-robust older adults. Understanding the relationship between the AGF and frailty in community-dwelling older adults may provide valuable insights into the multifaceted effects of frailty. Therefore, this study aimed to clarify the impact of frailty on the AGF in community-dwelling older adults.

## Methods

### Study design

This study used a cross-sectional design. As a basic research study, its primary objective was to clarify the impact of frailty on the AGF in community-dwelling older adults.

### Participants

Twenty-three community-dwelling older adults participated. The inclusion criteria were as follows: (1) aged ≥ 65 years, (2) living at home, (3) walking independently without assistive devices, (4) ability to visit the testing facility, and (5) a score of ≥ 24 on the Mini-Mental State (MMS), a Japanese cognitive screening tool comprising widely used items, including orientation, registration, attention, calculation, recall, and language [15]. The exclusion criteria were as follows: (1) prior experience with similar tasks and (2) a current or history of orthopaedic or neurological hand conditions affecting ADL. Participants were recruited from the Silver Human Resource Centre between August 2023 and March 2024. Those who agreed to participate in the study were selected as final participants after the study content was fully explained verbally and in writing. Based on a previous study [14] comparing the AGF of older and younger adults (effect size: Cohen’s d = 1.4), the sample size was calculated using G*Power with α = 0.05, 1-β = 0.8, and an effect size of 1.4, yielding 20 participants via a Mann–Whitney U-test. This study protocol was approved by the Ethics Review Committees of Mejiro University (approval no. 21 medicine−020) and Tokyo Metropolitan University (approval no. 2022002).

### Measurement items

#### Basic data

Basic data were collected, including age, sex, grip strength, MMS score, and Trail Making Test (TMT) results. The grip strength was measured twice, and the average was used. The dominant hand was determined using the Edinburgh Handedness Inventory, with a laterality index of ≥ 0 indicating right-handedness and < 0 indicating left-handedness [16].

#### Physical frailty

Frailty was assessed using the Japanese version of the Cardiovascular Health Study (J-CHS) criteria [17, 18]. The J-CHS includes five components: shrinking, low activity, exhaustion, weakness, and slowness. Shrinking was defined as weight loss of at least 2 kg in the past 6 months. Low activity was assessed based on whether the participant engaged in physical exercise or played sports at least once a week. Exhaustion was defined as feeling tired for no reason in the past 2 weeks. Weakness was defined as a decrease in grip strength of < 28 kg for males and < 18 kg for females. Slowness was defined as having a walking speed of < 1.0 m/s. Participants meeting ≥ 3 criteria were classified as frail, those meeting 1–2 as pre-frail, and those meeting no criteria as robust.

#### ADL

ADL was assessed using the Frenchay Activities Index (FAI), developed by Holbrook et al. [19]. This study used the validated Japanese self-administered version [20]. The FAI is a questionnaire-based assessment comprising 15 items related to applied ADLs and social activities, such as meal preparation, laundry, cleaning, hobbies, and travel. Participants were asked to respond to each item on a scale of 0 to 3 according to the frequency of the activity. The FAI assigns a total possible score of 45 across 15 items related to instrumental ADLs. As it is based on activity frequency, the FAI is a useful and simple clinical method for assessing living conditions.

#### AGF

The iWakka (Nagoya Institute of Technology) was used to evaluate AGF. The iWakka consists of a device, a control box, an iPad (Apple Inc., Cupertino, CA, USA), and the iWakka Viewer application installed on the iPad. The cylindrical device was made of a polyvinyl chloride (PVC) pipe (height: 80 mm, diameter: 65 mm). The PVC pipe was divided vertically into two parts, one end of which was fixed with a hinge on which intersecting leaf springs were attached. The strain on the plate springs caused by the opening and closing of the iWakka was measured using strain gauges, and the grasping force was plotted in real-time on a monitor to visualise changes in grasping force [12–14]. Participants adjusted their grasping force according to the target value displayed on the monitor. The absolute error between the target value and the actual grasping force was calculated and used as the AGF score. Importantly, AGF refers to the conceptual ability to adjust grasping force in accordance with task demands, whereas the AGF score is a numerical value indicating the magnitude of error. Therefore, a lower AGF score indicates better AGF, reflecting more accurate adjustment of grasping force.

The study used three types of sections: isometric sections, in which the grasping force remained constant over time; concentric sections, in which the grasping force gradually increased; and eccentric sections, in which the grasping force gradually decreased. A previous study of community-dwelling older adults living independently reported that AGF differed across sections, requiring different adjustment strategies for each [13]. Therefore, this study aimed to gain insight into the characteristics of adjustment tasks by calculating the AGF score (g) for all sections combined and for each section individually.

The measurement environment was set up as previously described [14]. The distance between the table and the body was approximately 10 cm, and the monitor was placed 50 cm from the edge of the table. To eliminate upper limb influence and maintain shoulder height, the iWakka was placed on an acrylic board with both elbows resting on the table and the forearm in a neutral rotation position to prevent tilting. Before measurement, participants performed a practice task with a target value different from that of the assessment tasks to understand the content of the tasks used in iWakka. The assessment task was then conducted under identical conditions. The dominant and non-dominant hands were tested in two trials each, and the average of both trials was used as the measurement value.

### Data analysis

Participants classified as pre-frail or frail by the J-CHS were assigned to the non-robust group, while those classified as robust were assigned to the robust group. A Mann–Whitney U test was conducted to assess differences in factors other than the AGF score (age, grip strength, MMS, TMT, and FAI) between the non-robust and robust groups. A chi-squared test examined differences in the male–female ratio between the groups. For the dominant hand ratio, Fisher’s exact test was used due to expected frequencies below five in some cells. A Mann–Whitney U test was then used to determine differences in AGF score between the non-robust and robust groups. Additionally, a Wilcoxon signed-rank test assessed differences in AGF scores between the dominant and non-dominant hands in each group. Statistical analysis was performed using IBM SPSS software (version 27) and a 5% significance level.

## Results

### Participant characteristics

The twelve participants in the robust group had a median (interquartile range [IQR]) age of 74.5 (69–76) years and comprised five males and seven females (Table 1). The median (IQR) grip strength was 25.1 (20.8–31.7) kg for the dominant hand and 22.1 (19.3–30.8) kg for the non-dominant hand. The median (IQR) MMS score was 28.0 (25.3–30.0) points, TMT-A was 46.5 (34.8–65.5) s, and TMT-B was 97.0 (88.3–123.5) s. Eleven participants were right-handed, and one was left-handed. The median (IQR) FAI was 32.0 (25.3–33.8). The median (IQR) AGF score for the dominant hand was 9.6 (7.7–13.9) g for total sections, 7.6 (4.4–9.3) g for isometric sections, 8.3 (6.0–17.2) g for concentric sections, and 11.8 (9.0–14.9) g for eccentric sections. For the non-dominant hand, the median (IQR) AGF score was 11.3 (7.8–14.0) g for total sections, 7.1 (4.0–10.3) g for isometric sections, 11.0 (9.1–20.5) g for concentric sections, and 11.3 (7.7–13.2) g for eccentric sections.

**Table 1:** Basic characteristics of the participants

		Robust group (*n* = 12)		Non-robust group (*n* = 11)	*p*
	*n*	Median (IQR)	*n*	Median (IQR)	
Age (year)		74.5 (69–76)		72.0 (70–76)	0.70
Sex, male/female	5/7		7/4		0.29
Grip strength (kg)					
Dominant hand		25.1 (20.8–31.7)		24.8 (20.5–27.3)	0.38
Non-dominant hand		22.1 (19.3–30.8)		23.3 (19.3–29.5)	0.93
MMS		28.0 (25.3–30.0)		29.0 (26.0–30.0)	0.41
TMT-A (s)		46.5 (34.8–65.5)		45.0 (38.0–63.0)	0.98
TMT-B (s)		97.0 (88.3–123.5)		108.0 (67.0–123.0)	0.61
Dominant hand, left/right	1/11		1/10		1.00
FAI		32.0 (25.3–33.8)		29.0 (27.0–35.0)	0.83
AGF score of the Dominant hand (g)					
Total sections		9.6 (7.7–13.9)		7.3 (6.3–11.3)	0.15
Isometric sections		7.6 (4.4–9.3)		5.1 (4.2–6.0)	0.24
Concentric sections		8.3 (6.0–17.2)		8.3 (5.6–12.0)	0.65
Eccentric sections		11.8 (9.0–14.9)		7.8 (7.1–13.9)	0.19
AGF score of the Non-dominant hand (g)					
Total sections		11.3 (7.8–14.0)		6.6 (6.0–8.3)	0.02
Isometric sections		7.1 (4.0–10.3)		4.0 (3.3–4.4)	0.04
Concentric sections		11.0 (9.1–20.5)		7.6 (5.7–8.3)	0.01
Eccentric sections		11.3 (7.7–13.2)		7.8 (6.5–10.4)	0.09

*IQR* Interquartile Range, *MMS* Mini-Mental State, *TMT* Trail Making Test, *FAI* Frenchay Activities Index, *AGF *Adjustability of Grasping Force

The non-robust group consisted of 11 participants with a median (IQR) age of 72.0 (70–76) years, including seven males and four females (Table 1). Eight participants met one J-CHS criterion, two met two criteria, and one met three criteria. In total, 43.5% were pre-frail, and 4.3% were frail. The median (IQR) grip strength was 24.8 (20.5–27.3) kg for the dominant hand and 23.3 (19.3–29.5) kg for the non-dominant hand. The median (IQR) MMS score was 29.0 (26.0–30.0) points, TMT-A was 45.0 (38.0–63.0) s, and TMT-B was 108.0 (67.0–123.0) s. Ten participants were right-handed, and one was left-handed. The median (IQR) FAI was 29.0 (27.0–35.0). The median (IQR) AGF score for the dominant hand was 7.3 (6.3–11.3) g for total sections, 5.1 (4.2–6.0) g for isometric sections, 8.3 (5.6–12.0) g for concentric sections, and 7.8 (7.1–13.9) g for eccentric sections. For the non-dominant hand, the median (IQR) AGF score was 6.6 (6.0–8.3) g for total sections, 4.0 (3.3–4.4) g for isometric sections, 7.6 (5.7–8.3) g for concentric sections, and 7.8 (6.5–10.4) g for eccentric sections.

The age (*p* = 0.70), sex ratio (*p* = 0.29), dominant hand grip strength (*p* = 0.38), non-dominant hand grip strength (*p* = 0.93), MMS score (*p* = 0.41), TMT-A (*p* = 0.98), TMT-B (*p* = 0.61), dominant hand ratio (*p* = 1.00), and FAI (*p* = 0.83) were not significantly different between the two groups (Table 1).

### Differences in AGF between the robust and non-robust groups

The non-robust group showed significantly lower AGF scores than the robust group in the total (*p* = 0.02), isometric (*p* = 0.04), and concentric (*p* = 0.01) sections of the non-dominant hand, indicating better AGF (Table 1). No significant differences were observed in the other sections.

### Comparison of AGF between dominant and non-dominant hands

In the robust group, AGF scores in the concentric section differed significantly between the dominant and non-dominant hands (*p* = 0.02), with the dominant hand showing a lower AGF score, indicating better AGF (Table 2). No significant differences were observed in the total (*p* = 0.31), isometric (*p* = 0.35), or eccentric (*p* = 0.39) sections.

**Table 2:** Comparison of AGF score between dominant and non-dominant hands

	Robust group			Non-robust group		
	Dominant hand (g)	Non-dominant hand (g)	*p*	Dominant hand (g)	Non-dominant hand (g)	*p*
Total sections	9.6 (7.7–13.9)	11.3 (7.8–14.0)	0.31	7.3 (6.3–11.3)	6.6 (6.0–8.3)	0.06
Isometric sections	7.6 (4.4–9.3)	7.1 (4.0–10.3)	0.35	5.1 (4.2–6.0)	4.0 (3.3–4.4)	0.04
Concentric sections	8.3 (6.0–17.2)	11.0 (9.1–20.5)	0.02	8.3 (5.6–12.0)	7.6 (5.7–8.3)	0.05
Eccentric sections	11.8 (9.0–14.9)	11.3 (7.7–13.2)	0.39	7.8 (7.1–13.9)	7.8 (6.5–10.4)	0.21

*AGF*: Adjustability of Grasping Force

In the non-robust group, AGF scores in the isometric section differed significantly between the dominant and non-dominant hands (*p* = 0.04), with the non-dominant hand showing a lower AGF score, indicating better AGF (Table 2). No significant differences were observed in the total (*p* = 0.06), concentric (*p* = 0.05), or eccentric (*p* = 0.21) sections.

## Discussion

### Participant characteristics

This study found no significant differences in the age, sex ratio, grip strength, MMS score, TMT results, dominant hand ratio, and FAI between the robust and non-robust groups. However, among participants in the non-robust group, eight met one J-CHS criterion, two met two criteria, and one met three criteria, indicating a higher proportion of pre-frail than frail individuals. Satake et al. [18] reported that, among community-dwelling older adults, 36.9% were robust, 51.9% were pre-frail, and 11.2% were frail. In contrast, this study found that 52.2% were robust, 43.5% were pre-frail, and 4.3% were frail, suggesting a relatively higher proportion of robust individuals than that in previous studies. This may be because participants were recruited from a Silver Human Resource Centre and were generally active, community-dwelling older adults who were independent in daily life.

Kaneno et al. [14] compared the AGF of young and older adults and reported that the AGF score in young adults was 4.4 g for both the dominant and non-dominant hands, while in older adults, it was 8.9 g for the dominant hand and 7.6 g for the non-dominant hand. The previous study [14] combined both robust and non-robust participants, whereas this study analysed them separately, allowing for a more detailed characterisation of the AGF in each group. In the robust group, the AGF score was 9.6 g in the dominant hand and 11.3 g in the non-dominant hand. Notably, the AGF score in the non-dominant hand in the robust group (11.3 g) was higher than that reported for older adults in the previous study (7.6 g) [14], indicating poorer AGF. In contrast, in the non-robust group, the AGF score was 7.3 g in the dominant hand and 6.6 g in the non-dominant hand, values higher than those typically reported in younger adults but generally lower than those in older adults, suggesting relatively better AGF. These findings provide insights into the AGF characteristics of the non-robust group. The AGF scores of the non-robust group closely matched those in previous studies, likely due to a high proportion of participants with similar characteristics of previous study.

### Relationship between frailty and AGF

This study compared AGF scores between the robust and non-robust groups and found that the non-robust group had significantly lower AGF scores in the non-dominant hand in the total (*p* = 0.02), isometric (*p* = 0.04), and concentric (*p* = 0.01) sections, indicating better AGF. However, no significant differences were observed in the AGF of the dominant hand between the two groups across all sections. A key finding was that significant differences between the two groups were only present in the AGF of the non-dominant hand. Yokoi et al. [21] reported that the non-dominant hand exhibited superior learning ability than the dominant hand in tasks requiring flexible adjustment of the opposite hand’s movement during two-handed movements. The non-robust group experienced some degree of physical function decline, meeting at least one of the J-CHS criteria, including shrinking, low activity, exhaustion, weakness, and slowness. To maintain daily routines despite declining physical function, individuals must adapt by optimising their remaining abilities. Previous studies have indicated that while younger adults predominantly use their dominant hand for daily tasks, older adults utilise both hands more equally due to a decline in dominant hand performance [22]. Therefore, the non-robust group, which encountered more situations requiring adaptation to age-related changes than the robust group, likely used their non-dominant hand more frequently. The high learning ability for adjustment tasks of the non-dominant hand may have facilitated greater AGF. Additionally, previous studies have reported that older adults are often unaware of the age-related decline in dominant hand superiority [22]. This highlights the need to increase older adults’ awareness of AGF changes associated with ageing. Furthermore, unlike previous hand function evaluation methods, the iWakka device used in this study detected fine force adjustments during task execution. This capability likely enabled the identification of frailty-related characteristics not previously reported. These findings suggest that AGF assessment may serve as a sensitive marker of early functional change and may be useful in clinical screening or rehabilitation planning for older adults at risk of frailty.

This study found no significant differences in age, male/female ratio, hand strength, MMS score, TMT results, dominant hand ratio, and FAI between the two groups. Isa et al. [23] reported that monkeys with spinal cord injuries restore motor function by disinhibiting the brain and reconstructing new neural circuits. Similarly, Morita et al. [24] investigated age-related changes in brain inhibition using a one-handed motor task and found that ipsilateral motor cortex inhibition increases with growth but declines with age. This suggests that the ipsilateral motor cortex may facilitate new neural circuit formation to compensate for age-related declines in brain function. Given that physical function and daily activity performance did not differ significantly between the two groups, the AGF in the non-dominant hand may serve as an adaptive mechanism compensating for frailty-related functional decline. Frailty involves multifaceted changes, including physical, mental/psychological, and social frailty, necessitating adaptations across multiple domains. This study suggests that the AGF in the non-dominant hand contributes to this adaptive process. Besides AGF, other compensatory or adaptive mechanisms may counteract frailty-related changes, requiring further research. These findings may indicate the involvement of adaptive neuroplastic changes; however, neurophysiological evidence supporting such compensatory mechanisms in frail older adults is currently limited. Accordingly, while the enhancement of AGF in the non-dominant hand observed in this study could be attributed to neuroplastic adaptation, this interpretation should be considered with caution, and further empirical validation is warranted.

Additionally, in the non-robust group, the AGF was better in the non-dominant hand than in the dominant hand during isometric sections. Kaneno et al. [13] reported similar findings in community-dwelling older adults living independently, where AGF was greater in the non-dominant hand during eccentric sections. Conversely, in the robust group, AGF was better in the dominant hand during the concentric section. This difference may result from the greater physical function reserves of the robust group, which reduce reliance on the non-dominant hand for compensation. By separately analysing the robust and non-robust groups, this study provided more detailed insights into the AGF characteristics in each group.

### Limitations of this study and future challenges

This study investigated the influence of frailty on the AGF in community-dwelling older adults. However, some limitations must be acknowledged.

First, the sample size was small. Participants were classified into robust and non-robust groups, but only one participant met the criteria for frailty. Therefore, subgroup analyses distinguishing between prefrail and frail individuals were not feasible.

Second, participants were recruited from a Silver Human Resources Centre, which may have introduced selection bias towards active older adults who were independent in daily life. This likely contributed to the low proportion of individuals who met the frailty criteria. Accordingly, the findings of this study are most applicable to community-dwelling older adults with relatively high functional capacity and may not be generalisable to institutionalised or clinically frail populations. Future studies should aim to include older adults with a broader range of physical and cognitive functions and adopt more diverse recruitment strategies. A larger and more representative sample would enhance external validity and enable more meaningful subgroup comparisons.

Third, although the FAI was used to assess general activity levels, the study did not collect information on factors that may affect AGF, such as hand-use patterns, occupational history, or bimanual activities (e.g., hobbies). Future research should incorporate detailed assessments of manual activity history, changes in hand dominance, and fine motor hobbies. These additions would help distinguish the effects of frailty from those of prior behavioural patterns or compensatory adaptations.

Fourth, cognitive functions, including executive function as assessed by the TMT-B, may have influenced AGF. Although we considered including these variables in a multivariate analysis, we refrained from conducting the analysis owing to the small sample size and the associated risk of overfitting. Future studies should use larger samples to allow for appropriate statistical adjustment for such cognitive factors.

Finally, as a basic research study, the primary aim was to describe AGF rather than to identify specific influencing factors. Future studies are needed to clarify the underlying factors and physiological mechanisms associated with AGF, thereby deepening our understanding of motor adaptation in the context of ageing and frailty progression.

## Conclusions

This study demonstrated that non-robust older adults exhibited superior AGF in the non-dominant hand compared to robust individuals. These results suggest a potential compensatory adaptation to functional decline. AGF assessment may serve as a sensitive marker for early detection of functional changes associated with frailty in older adults.

## Data Availability

The data that support this study’s findings are available from the corresponding author on reasonable request. The data are not publicly available due to ethical restrictions.

## Abbreviations

ADL: Activities of daily living
AGF: Adjustability of grasping force
FAI: Frenchay Activities Index
MMS: Mini-Mental State
TMT: Trail Making Test
